# An Evaluation of Antifungal Agents for the Treatment of Fungal Contamination in Indoor Air Environments

**DOI:** 10.3390/ijerph120606319

**Published:** 2015-06-02

**Authors:** Senthaamarai Rogawansamy, Sharyn Gaskin, Michael Taylor, Dino Pisaniello

**Affiliations:** 1 Occupational and Environmental Hygiene Laboratory, Discipline of Public Health, School of Population Health, University of Adelaide, South Australia 5005, Australia; E-Mails: Sentha.Rogawansamy@unisa.edu.au (S.R.); michael.taylor@flinders.edu.au (M.T.); dino.pisaniello@adelaide.edu.au (D.P.); 2 Health and Environment, School of the Environment, Flinders University, South Australia 5042, Australia

**Keywords:** Airborne fungi, indoor air quality (IAQ), vinegar, tea tree oil, inhibition zone

## Abstract

Fungal contamination in indoor environments has been associated with adverse health effects for the inhabitants. Remediation of fungal contamination requires removal of the fungi present and modifying the indoor environment to become less favourable to growth.  This may include treatment of indoor environments with an antifungal agent to prevent future growth. However there are limited published data or advice on chemical agents suitable for indoor fungal remediation. The aim of this study was to assess the relative efficacies of five commercially available cleaning agents with published or anecdotal use for indoor fungal remediation. The five agents included two common multi-purpose industrial disinfectants (Cavicide^®^ and Virkon^®^), 70% ethanol, vinegar (4.0%−4.2% acetic acid), and a plant-derived compound (tea tree (*Melaleuca alternifolia*) oil) tested in both a liquid and vapour form. Tea tree oil has recently generated interest for its antimicrobial efficacy in clinical settings, but has not been widely employed for fungal remediation. Each antifungal agent was assessed for fungal growth inhibition using a disc diffusion method against a representative species from two common fungal genera, (*Aspergillus fumigatus* and *Penicillium chrysogenum*), which were isolated from air samples and are commonly found in indoor air. Tea tree oil demonstrated the greatest inhibitory effect on the growth of both fungi, applied in either a liquid or vapour form. Cavicide^®^ and Virkon^®^ demonstrated similar, although less, growth inhibition of both genera. Vinegar (4.0%–4.2% acetic acid) was found to only inhibit the growth of *P. chrysogenum*, while 70% ethanol was found to have no inhibitory effect on the growth of either fungi. There was a notable inhibition in sporulation, distinct from growth inhibition after exposure to tea tree oil, Virkon^®^, Cavicide^®^ and vinegar. Results demonstrate that common cleaning and antifungal agents differ in their capacity to inhibit the growth of fungal genera found in the indoor air environment. The results indicate that tea tree oil was the most effective antifungal agent tested, and may have industrial application for the remediation of fungal contamination in residential and occupational buildings.

## 1. Introduction

Populations in developed countries spend more than 90% of their time indoors and the installation and maintenance of HVAC (heating, ventilating, and air conditioning) systems has become increasingly important. Fungal spores are common components of both indoor and outdoor air. However, fungi have become one of the leading causes of indoor air quality (IAQ) complaints in occupational settings [1,2,3]. Fungi are now frequently implicated as a causative agent in “sick building syndrome” [4] and fungal contamination of indoor environments has been linked to adverse health effects including headache, allergy, asthma, irritant effects, respiratory problems, mycoses (fungal diseases), and several other non-specific health problems [5]. More than 80 genera of fungi have been associated with symptoms of respiratory tract allergies with *Cladosporium, Alternaria, Aspergillus* and *Penicillium* amongst the most common allergenic genera [6]. Beyond allergenicity, many fungi produce microbial volatile organic compounds (MVOCs) and mycotoxins that are believed to irritate the respiratory system. Research from animal studies and data from occupational settings have shown that exposure to mycotoxins can result in mucus membrane irritation, skin rashes, dizziness, nausea, and immunosuppression [7]. 

Fungi are able to grow in indoor environments where there is sufficient moisture and a nutrition source, such as wood, paint and insulation and release spores as part of their reproductive process [8]. Both temperature and water availability affect growth and sporulation characteristics of airborne fungi, with higher ambient temperatures and available water favouring faster growth. Release of spores is subsequently increased by intermittent periods of dryness where spores are dispersed, and moisture allowing for further growth and sporulation. Fungal species found indoors usually reflect those in the outdoor environment, although concentrations may change seasonally or locally where the indoor environment is favourable to the growth of particular species [3]. The most common genera are saprophytes, including those living on decaying plant material, *Cladosporium*, *Alternaria*, *Epicoccum* and *Aureobasidium*, while soil-based species, such as *Aspergillus* and *Penicillium*, are relatively low in number in outdoor air but are found at increased levels indoors [9]. *Aspergillus* sp. and *Penicillium* sp. have been recognized as significant indoor air allergens [10]. Fungi typically enter a building through heating, air conditioning and ventilation systems, windows, doors, and as contaminants on building materials. Prolonged high moisture levels in a building then provide the necessary conditions for fungal growth and sporulation to occur and mechanical disturbances can cause the spores to become airborne.

In Australia, there are currently no indoor air quality guidelines for fungi in air. However, there are a number of international guidelines available, both current and historic (as outlined in [3]) but typically World Health Organization guidelines are adopted [11]. As the relations between dampness, microbial exposure and health effects cannot be quantified precisely, “no quantitative health-based guideline values or thresholds can be recommended for acceptable levels of contamination with microorganisms” [11]. Instead, it is recommended that dampness and mould related problems be prevented and rapidly remediated when they occur to reduce the risk of hazardous exposure to microbes and chemicals. Other considerations include the relationship between indoor and outdoor levels as represented by simultaneously collected samples. The principle is that lower indoor than outdoor fungal levels indicate an acceptable indoor environment and the diversity of indoor fungal genera should be similar to that found outdoors [3,5].

To minimise the potential for exposure, it is essential to remediate an indoor space with visible fungal contamination. The remedial process involves the removal of visibly contaminated building material and the use of an antifungal product to treat surfaces, in conjunction with steps to modify the indoor environment to prevent future fungal growth [12]. The use of a HEPA vacuum cleaner is recommended in combination with damp wiping non-porous surfaces to remove the dispersed spores in buildings [13].

An antifungal agent, or fungicide, is a biocidal chemical compound or biological organism used to kill or inhibit fungi or fungal spores. The Australian Mould Guideline [14] is commonly adopted by industry and recommends damp wiping with a detergent, vinegar solution or alcohol solution for removing fungi from contaminated surfaces. It also lists antifungal agents such as bleach, alcohol (100%), quaternary ammonium compounds and formaldehyde as chemicals that are used in the treatment of fungi on surfaces but does not explicitly recommend the use of these agents for preventing future growth. 

Fungicides perceived to be of “natural” origin are being investigated increasingly for use in fungal contamination remediation. Vinegar (acetic acid) is reported to have an antimicrobial effect on fungi in various applications. Vinegar vapour application has been demonstrated to prevent the germination of conidia of fruit decay fungi *Penicillium expansum, Monilinia fructicola* and *Botrytis cinerea* in strawberries, apples, and stone fruit [15] and *Colletotrichum coccodes* in tomato fruit [16]. Vinegar in water, in an undefined concentration, has been recommended by the Australian Mould Guidelines [14], for damp wiping hard surfaces in the remediation of an indoor fungal contamination. There is also growing interest in the antimicrobial efficacy of compounds such as essential oils. Tea tree oil (TTO) is purported to be a plant-derived antifungal agent that utilises the same mechanism of toxicity in fungi as 70% ethanol and vinegar, by killing the conidia and preventing germination of the spores [17,18]. There are some studies investigating the antifungal efficacy of TTO in the clinical setting: TTO has been used in the treatment of fungal infections such as vaginal and oral candidiasis and has shown strong antiviral activity on the Influenza A virus and *E.coli* Phage M13 Phage [19]. Tea tree oil has not been widely considered for environmental application beyond agricultural use, and there is limited information on its use in indoor fungal contamination [12,20]. 

The aim of this study was to assess the relative efficacies of five commercially available cleaning agents with published or anecdotal use for indoor fungal remediation. The five agents included two common multi-purpose industrial disinfectants (Cavicide^®^ and Virkon^®^), 70% ethanol, vinegar (4.0%–4.2% acetic acid), and a plant-derived compound (tea tree (*Melaleuca alternifolia*) oil). 

## 2. Experimental Section 

### 2.1. Antifungal Agent Selection

The antifungal agents used in this study are commercially available and commonly used products. Virkon^®^ and Cavicide^®^ are broad spectrum disinfectants currently used to sanitise surfaces and equipment in the medical and healthcare sector. Virkon^®^ (Antec International Limited) is the brand name for a disinfectant containing peroxygenic acid (50% potassium monoperoxysulphate, potassium hydrogen sulphate and potassium sulphate) and is applied at a concentration of 1% for 10 min and claims to kill bacteria, viruses, fungi and spores. However, there is limited published information on Virkon’s^®^ capacity to kill fungi and spores. For this study, Virkon^®^ was prepared at a range of test concentrations: 10%, 5%, 3% and 1%. Cavicide^®^ (17.2% Isopropyl Alcohol, 0.28% Benzethonium Chloride; Bacto Laboratories Pty Ltd) is a broad spectrum disinfectant used undiluted for the cleaning of hard surfaces of medical and dental devices and claims to have bactericidal, virucidal, fungicidal and tuberculocidal properties. Cavicide^®^ was assessed undiluted and at a concentration of 75%. Tea tree oil (100% *Melaleuca alternifolia* oil; Bosisto’s Pty Ltd) is commonly used as a “natural” topical antiseptic and also as a complementary medicine for fungal infections. Tea tree oil was tested both as a direct contact solution and in vapour phase given evidence in clinical settings of its potential antifungal effects in both forms, and growing interest in the antimicrobial effects of this “natural” product [12,17,18]. Vinegar (4.0%–4.2% acetic acid) and 70% ethanol were also chosen due to their common recommendation in fungal remediation and anecdotal support in disinfection of hard surfaces. 

### 2.2. Environmental Fungi Sampling and Identification

Environmental air samples were collected onto malt extract agar (MEA) plates using a BioStage® single-stage viable cascade impactor, attached to a SKC QuickTake™ 30 Air sampler. Air samples were collected for 2 min each at a flowrate of 28.3 L/min [21]. Agar plates were incubated for 7 days at 25 °C. After 7 days, fungal colonies were tape lifted onto glass slides and stained with lactophenol cotton blue for 5 min before observation by phase contrast microscopy (Nikon Eclipse C*i*; Coherent Scientific). Two genera representing the most commonly isolated fungi from environmental samples were selected for use in the study (an *Aspergillus fumigatus* from indoors, and *Penicillium chrysogenum* from outdoors). The fungi selected for use in this study represent extremely common airborne fungi, capable of growth on a wide range of substrates, and frequently isolated from indoor environments not only in Australia but worldwide [22,23].

To obtain pure cultures, three agar plugs 6 mm in diameter were cut from the edge of an individual *A. fumigatus* colony using the end of a glass Pasteur pipette and aseptically transferred to a fresh MEA plate and evenly placed apart. This was repeated for *P. chrysogenum*. The plates were wrapped in parafilm and incubated at 25 °C for 7 days and observed for the appearance of pure fungal colonies.

### 2.3. Antifungal Efficacy Using Disc Diffusion Assay

Ficker’s [24] disc diffusion assay was used to assess the inhibitory effect of antifungal agents on the growth of *A. fumigatus* and *P. chrysogenum*. Spore suspensions were prepared by flooding fungal culture plates with 3 mL of sterile distilled water and a sterile loop was used to agitate colonies. One hundred microlitres of each spore suspension was used to inoculate MEA plates which were left to dry at room temperature for 15 min. Inoculated MEA plates were sectioned into halves and 20 µL of each of the test agents were pipetted onto an autoclaved Whatman^TM^ filter paper disc 9 mm in diameter and placed in the middle of each section. 

Phenol (88% solution) was used as a positive control for fungal growth inhibition and sterile distilled water was used as a negative control. Plates were sealed with parafilm and incubated at 25 °C for 7 days before observation for fungal growth and formation of inhibition zones around disks. Each test was repeated a minimum of 3 times for each agent (additional replicates n = 4−6 were performed where a positive result was found). An agent was categorised as having antifungal activity when the diameter of the inhibition zone was larger than 9.5 mm, 0.5 mm larger than the diameter of the paper disc [24]. This value serves as a frame of reference against which the antifungal susceptibility of the fungi can be compared. The larger the concentric area of inhibited growth, the greater the efficacy of the antifungal agent.

The antifungal activity of TTO in vapour phase was assessed using a modified method of [25]. A 100 µL spore suspension of each fungal species was spread onto fresh MEA plates and allowed to air dry for 15 min at room temperature. Twenty microliters of TTO was pipetted onto a 9 mm paper disc and placed on the inner surface of the petri dish lid, having no direct contact with the surface of the inoculated agar. Plates were rapidly sealed in parafilm to minimise escape of the volatile components, then incubated at 25 °C and observed after 7 and 14 days. Antifungal efficacy was determined by measuring the mean perpendicular diameter of the inhibition zone. To examine the growth inhibitory effect of TTO over time, the TTO vapour and solution disc assay plates for both fungal genera were re-incubated for an additional seven days after initial treatment, bringing the total incubation period to 14 days. Following re-incubation, the assay plates were re-measured for growth inhibition.

### 2.4. Data Analysis

Comparisons of inhibition zones by antifungal agents and controls were performed using one-way ANOVA. Assumption of data normality were checked and met for parametric analysis. Post-hoc analysis was performed where required using Tukey Multiple Comparisons test. Significance for all tests was set at *p* ≤ 0.05. Statistical analyses were performed using SPSS v.16 and GraphPad Prism V.4 software.

## 3. Results and Discussion

**Figure 1:**
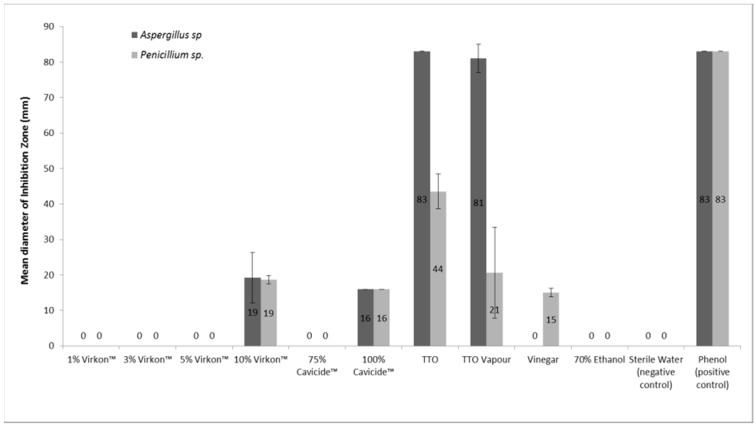
Diameter of growth inhibition zones of *Aspergillus fumigatus* and *Penicillium chrysogenum* after treatment using various antifungal agents. Mean ± SD (N = 40).

Tea tree oil applied as a direct contact solution was found to have the highest inhibitory effect on the growth of both *A. fumigatus* and *P. chrysogenum* after a seven-day incubation period compared to the other antifungal agents tested (Figure 1). *A. fumigatus* growth was completely inhibited by TTO with a mean inhibition zone diameter (83 mm) significantly greater than other test compounds (*p* < 0.0001, R^2^ = 0.9761) and comparable to the results of the positive control phenol. TTO was less toxic to the *P. chrysogenum* (mean inhibition zone diameter of 43.5 mm ± 4.93), but more effective at inhibiting growth than the other compounds tested (*p* < 0.001, R^2^ = 0.9620). TTO when applied in vapour form, was found to be less effective than direct application in inhibiting the growth of condidate fungi (*A. fumigatus* mean inhibition zone diameter of 81 mm ± 4; *P. chrysogenum* mean inhibition zone diameter of 20.6 mm ± 12.85 for the vapour assay).

Virkon^®^ was only effective at reducing fungal growth at a concentration of 10%, demonstrating a mean inhibition zone diameter of 19.25 mm (± 7.08) for *A. fumigatus,* and 18.67 mm (± 1.15) for *P. chrysogenum* (Figure 1). Five percent, 3%, and 1% Virkon^®^ solutions had no effect on the growth of either fungi.

Undiluted Cavicide^®^ was found to have a similar inhibitory effect on the growth of both fungi, with a mean inhibition zone diameter of 16 mm (± 0) for both (Figure 1). In contrast, 75% Cavicide had no inhibitory effect on the growth of either fungi.

Vinegar (4.0%–4.2% acetic acid) had an inhibitory effect on the growth of *P. chrysogenum* with a mean inhibition zone diameter of 15 mm (± 1.15), but did not show an inhibitory effect on the growth of *A. fumigatus* (Figure 1). Seventy-percent ethanol had no visible effect on the growth of either fungi (Figure 1).

For some test agents, although growth remained unaffected, inhibition of sporulation was noted. Five percent, 3%, and 1% Virkon^®^ inhibited sporulation of *P. chrysogenum*, but had no visible effect on the sporulation of *A. fumigatus*. Seventy-five percent Cavicide^®^ inhibited the sporulation of *A. fumigatus* but not *P. chrysogenum*. Vinegar suppressed sporulation of *P. chrysogenum* but had no effect on the sporulation of *A. fumigatus*.

**Figure 2:**
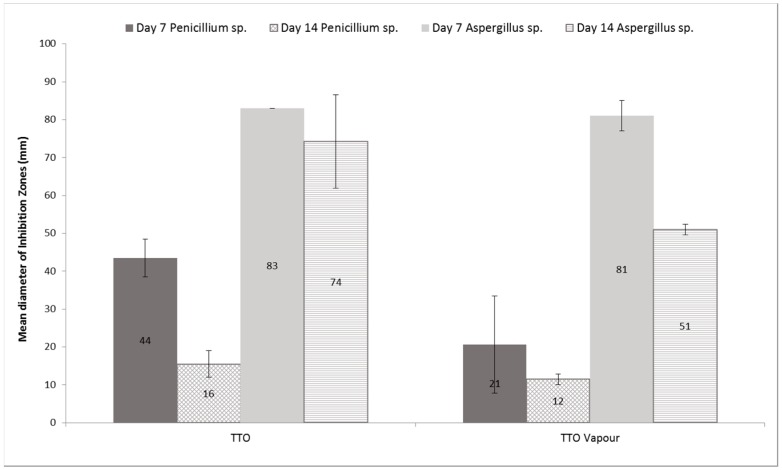
Difference in growth inhibition zones of *Aspergillus fumigatus* and *Penicillium chrysogenum* 7 and 14 days after initial treatment with tea tree oil. Mean ± SD (N = 24).

The potential longer-lasting growth inhibition effects of TTO on *A. fumigatus* and *P. chrysogenum* are shown in Figure 2. TTO applied as a solution became less effective at inhibiting growth of *P. chrysogenum* after 14 days of treatment (*p* = 0.004), but not so for *A. fumigatus*. TTO vapour showed decreased growth inhibition as a function of time against both fungi, although only shown to be significant for *A. fumigatus* (*p* = 0.0002). Sporulation was observed on day 14 of treatment on all TTO exposed cultures. 

The results of this study indicate that common cleaning and antifungal agents differ in their capacity to inhibit the growth of common indoor fungal genera. In fact, strains within a species may behave differently to biocides, as demonstrated by Tortorano *et al*. [26] for fifteen *Aspergillus fumigatus* clinical isolates. The broad spectrum disinfectant Virkon^®^ did show evidence of antifungal activity against both genera, but only at the highest test concentration (10%), which is ten times the manufacturer’s recommended concentration for disinfecting surfaces. Five percent, 3%, and 1% Virkon^®^ solutions had no effect on the growth of either fungi, a result which is corroborated by [27] who assessed the *in vitro* efficacy of 1% Virkon against bacteria, fungi, viruses and spores. Hernandez *et al*. [27] demonstrated bactericidal activity against both Gram-positive and Gram-negative vegetative bacteria in 5 min, virucidal activity was shown against poliovirus and biocidal activity was shown against *C.albicans* after 15 min. However, there was no fungicidal activity against *Penicillium verrucosum* and *Absidia corymbifera* and *Bacillus cereus* spores even after 1 h of contact. The authors concluded that 1% Virkon^®^ is a low level disinfectant as it has a rapid biocidal effect against vegetative bacteria and viruses but is incapable of killing endospores and fungi within a reasonable amount of time. Virkon^®^ was also found to be ineffective at disinfecting *Mycobaterium tuberculosis* at both 1% and 3% concentrations on hospital instruments and surfaces [28]. Broadley *et al*. [29] reported that at 2%, 3%, and 4% concentrations, Virkon^®^ was unable to provide a satisfactory kill of mycobacteria, but retarded the onset of growth, and was not recommended as a mycobactericidal agent. The chemical composition of Virkon^®^ includes a stabilised blend of peroxy compounds, surfactant, organic acids and an inorganic buffer system. Its mechanism of action is considered to be the denaturation of cellular proteins through its high oxidizing activity [30]. Although Virkon^®^ at 10% concentration was shown to be somewhat effective as an antifungal agent in the current study, it could pose potential hazards to workers or occupants due to its corrosivity and toxicity at such a high concentration and would have limited application in the indoor air environment. 

Cavicide^®^, another broad spectrum disinfectant, similarly demonstrated some antifungal activity against both genera in the current study, but only when applied undiluted. No studies assessing the antifungal activity of Cavicide^®^ have previously been reported. Some evidence for antibacterial activity has been reported in the literature, for example it was effective in lowering *Staphylococci* bacterial loads on bed rails in a hospital by up to 97% [31], which was attributed to its high alkaline nature, but was found to be ineffective against *Bacillus subtilis* [32]. Cavicide^®^ is composed of isopropanol and diisobutyl phenoxyethoxyethyl dimethyl benzyl ammonium chloride. It is used undiluted in laboratories and hospitals for surface and instrument disinfection. It has been reported to be 50% more effective in lowering the microbial load on patient bed rails in comparison to disinfectants containing quaternary ammonium compounds [31], although within six hours of application, the microbial load had exceeded acceptable levels, indicating that the frequency of application is an important variable for maintaining low microbial loads [31]. The antimicrobial efficacy of Cavicide^®^ may be associated with its high alcohol concentration in conjunction with a pH of 12. As demonstrated for Virkon^®^, it seems the application of Cavicide^®^ to the indoor air environment has limited viability due its chemical composition, despite Cavicide^®^ being able to reduce fungal growth at the recommended concentration in both genera.

The Australian Mould Guidelines [14] recommend the use of vinegar or alcohol for the removal of mould from contaminated surfaces. However, this study demonstrates that vinegar has limited antifungal action while ethanol (70%) is ineffective as an antifungal agent for the treatment of two common fungal genera in the indoor air environment. Vinegar (4.0%–4.2% acetic acid) was found to inhibit the growth of *P. chrysogenum* but not *A. fumigatus* in the current study. Vinegar is a known antimicrobial agent and there is some evidence to suggest that it possesses antifungal properties. Sholberg *et al*. [15] found that vinegar vapour effectively inactivated the conidia of several decay fungi on fruit due to its acetic acid content (5%), by lowering the pH of the cell protoplasm and killing the conidia. In another study, bamboo vinegar was found to have a dose-dependent inhibitory effect on the growth of bacteria and fungi due to its active compounds phenols, acetic acid and alcohols [33]. The use of vinegar as a fungal remediation agent may be warranted, however its lack of persistence on surfaces may limit its use to removal of fungal contamination on non-porous materials and prevent future growth. An important avenue of future work is to explicitly test the application of cleaning agents to a range of surface materials. Agar is used in virtually all laboratory studies as a model for many real world conditions, however it is not without limitations. Due to its high water content it may exert some degree of dilution in highly water-soluble compounds. Similarly, chlorine based disinfectants may be quenched by the organic content of the media. However, these effects are also likely to be noted in building materials or other test matrices, and further characterisation of compounds in real world conditions is warranted.

Ethanol is widely used for general surface disinfecting and has reported biocidal efficacy against bacteria, fungi and viruses in the concentration range of 50%–90% [34]. In the current study, 70% was found to be completely ineffective as an antifungal agent against common airborne fungal genera. In contrast, in the food industry, ethanol has been shown to inhibit mould growth on bread, which is usually spoiled by *Penicillium*, *Aspergillus*, and *Cladosporium* [35]. It has also been used to prevent postharvest decay of fruits [15,36]. Ethanol vapour was found to inhibit germination of the fungal conidia (*Penicillium chrysogenum*) isolated from pastry products, but this was found to be reversible over time as some spores remained viable [37]. Ethanol interacts with cellular membranes increasing membrane permeability and causing leakage of solutes and cell lysis. Higher concentrations of ethanol are required to kill fungal spores than bacteria, which show a maximum kill efficacy of 70% ethanol [34]. Dao *et al*. [38] found that ethanol as both a liquid and vapour could significantly inactivate fungal spores (*Penicillium chrysogenum*, *P. digitatum*, and *P. italicum*) and recommended further investigation into the use of ethanol vapour in place of ethanol solution to prevent mould growth in workplaces. 

Tea tree oil as a direct contact solution, was the most effective at inhibiting fungal growth of both test species among all the agents assessed in the current study. This result is in agreement with previously published data on the antimicrobial efficacy of TTO *in vitro* from a clinical setting [17,18]. The mechanism of TTO’s antifungal action is believed to be by the alteration of the cell membrane structure, causing it to become permeable, which leads to the leakage of cellular material and disruption to cellular functions [39]. Hammer *et al.* [17] found that TTO had both an inhibitory effect and a fungicidal effect on filamentous fungi. The authors found both germinated conidia and non-germinated conidia of the fungal isolates demonstrated susceptibility to TTO. In the current study, results show that *P. chrysogenum* is less susceptible to TTO than *A. fumigatus*. The reduced susceptibility of different conidia to antifungal agents is possibly due to the thickness, composition and density of the conidial wall. 

Furthermore, tea tree oil was found to be more effective as a direct contact solution at inhibiting the growth of *A. fumigatus* and *P. chrysogenum*, than in vapour phase. This challenges previous reports of TTO in vapour phase having a greater inhibition effect than TTO in solution on fungal growth [40]. Shao *et al*. [41] also reported that TTO vapour displayed a greater inhibitory effect on fungal growth than in direct contact. Soylu *et al*. [25] proposed that TTO vapour may be more readily absorbed by fungal mycelium since it is not diluted by the water content of agar medium as per the direct contact phase in solution. The main compounds reported to be responsible for the antimicrobial activity of TTO are terpinen-4-ol and 1,8-cineole [41]. TTO was found to exhibit antifungal activity in contact and vapour phase on the mycelial growth of *B.cinerea* by rupture of the cell wall and by increasing membrane permeability [41]. Hammer *et al.* [17] found that TTO had both an inhibitory effect and a fungicidal effect on filamentous fungi including *Aspergillus niger, Aspergillus fumigatus* and *Penicillium spp* at Minimum Inhibitory Concentrations of 0.06%–0.12% (v/v) and Minimum Fungicidal Concentrations (MFC) of 2%–8% (v/v). Both germinated conidia and non-germinated conidia of the isolates demonstrated susceptibility to TTO. Time-kill assays showed that the duration of exposure of the fungi to TTO could influence the fungicidal action. However, there is limited information on TTO in indoor applications. Tea tree oil was the overall most effective antifungal agent and could be explored for remediation of fungal contamination. Consideration should be given to any potential health effects for occupants from exposure to TTO by direct contact (dermal) with residue or inhalation of vapour. TTO constituents may have skin sensitizing properties (e.g., limonene), although scientific evidence regarding the inhalational health effects of these aromatic compounds remains limited [17,42].

Suppression of sporulation as distinct from growth was a notable occurrence in the current study. Sporulation inhibition is not typically reported in the literature as an antimicrobial effect, but may still be considered a form of remediation as inhibition of spore formation would be expected to reduce the ongoing inoculum potential and generation of allergenic particles from fungi. Carson *et al.* [18] and Inouye *et al.* [43] reported the ability of TTO vapour to inhibit fungal growth and affect sporulation. Inouye *et al.* [44] reported that TTO vapour could affect fungal sporulation by a direct absorbing effect on aerial hyphae and demonstrated that sporulation inhibition was an effect of inhibition of respiration rather than the inhibition of growth. Similarly, in a study of essential oil effects on fungal cultures, some plates showed no growth inhibition whilst sporulation was completely inhibited with TTO [40], demonstrating the greatest inhibitory effect on sporulation of the three compounds tested. While only 10% Virkon^®^ displayed a growth inhibition effect, all concentrations of Virkon^®^ were found to have suppressed the sporulation of *P. chrysogenum* in the current study. Similarly, both test concentrations of Cavicide^®^ were found to inhibit the sporulation of *A. fumigatus*. In contrast, vinegar was found to only inhibit the growth and sporulation of *P. chrysogenum* Antifungal agents displaying inhibition of sporulation with limited growth inhibition effect should not necessarily be discounted as effective antifungal agents. Sporulation inhibition could provide an important benefit in fungal remediation by reducing contamination persistence and by reducing the ongoing allergenicity of fungi present in indoor environments. 

There is limited published information more broadly on the use of antifungal agents in indoor fungal contamination. Chakravarty and Kovar [12] tested five antifungal agents, Sanimaster^®^, 17% hydrogen peroxide, 70% isopropyl alcohol, bleach and Sporicidin^®^ used in indoor fungal remediation by companies in the USA. They studied the inhibitory effects of growth and spore germination of six fungal species commonly found indoors; *Alternaria alternata*, *Aspergillus niger*, *Chaetomium globosum*, *Cladosporium herbarum*, *Penicillium chrysogenum* and *Stachybotrys chartarum.* The six species were inoculated into pine wood blocks and incubated at 25 °C. There was a significant inhibitory effect on growth and spore germination exhibited by all five compounds tested within 12 h of treatment. However, when the agents had been rinsed off with distilled water, the fungal spores recovered and became viable after a 24-h incubation period. Two weeks after the treatment, fungal growth was found to be entirely uninhibited. This reversible inhibitory effect is described as mycostasis, in which the growth of the spores is inhibited by the antifungal compounds without any effect on viability. The authors concluded that most antifungals are effective on hard non-porous surfaces but viable spores within porous surfaces may be unaffected and become dormant when an antifungal is applied. Huang *et al.* [20] found that TTO applied on the filter surface of a HVAC system inactivated environmental fungal spores and prevented the re-entry of the dead spores back into the air by adhesion. There could be potential for the use of TTO on HVAC filters to control bioaerosol concentrations in occupational and residential settings and is classified as ‘generally regarded as safe’ (GRAS) by the United States Food and Drug Administration (FDA). 

A 2.4% sodium hypochlorite (NaOCl) treatment was tested on *Alternaria alternate*, *Aspergillus niger*, *Cladosporium herbarum*, *Penicillium chrysogenum*, *Stachybotrys chartarum* and *Trichophyton mentagrophytes* and found to inactivate all the spores of the stock cultures to undetectable levels after 5 min contact time on non-porous surfaces and after 10 min contact time on porous surfaces [45]. These results suggest that hypochlorite disinfectants are effective in the reduction of fungal proliferation and allergen levels in the indoor environment. Sodium hypochlorite has also been recommended for use in very low concentrations (0.04%) to inactivate fungi on grains, nuts and vegetables [46].

This work broadly highlights the need for consistency in advice given for remediation of fungal damage and the importance of verifying anecdotal evidence or “common knowledge advice” of antifungal agents and cleaning procedures. Importantly, any adopted antifungal agent would need to be implemented in conjunction with technical measures, such as improving ventilation and reducing humidity in the indoor air to prevent future growth. 

## 4. Conclusions 

This study assessed the efficacy of different antifungal agents on two commonly found fungal genera from the indoor air environment. Ideally, an effective antifungal agent would inhibit the growth of a range of common fungal genera, whilst increasingly less effective antifungal agents would only inhibit sporulation or inhibit the growth of a limited range of fungal genera. Some antifungal agents assessed in this study completely inhibited growth while others had reduced or no visible effect. The best performing agents were tea tree oil in liquid and vapour applications, undiluted Cavicide^®^ and 10% Virkon^®^ which were effective in inhibiting the growth of both genera. However, Cavicide^®^ and 10% Virkon^®^ may have limited viability for use in the indoor air environment due to their chemical composition. Some agents were only effective against one species and not the other, such as vinegar, which only inhibited the growth and sporulation of *P. chrysogenum*. The use of vinegar as an antifungal agent may not be effective against the diverse fungal genera found in the indoor air environment. Seventy percent ethanol had no inhibitory effect on either fungi and is not suggested as an effective antifungal treatment. Some agents completely inhibited sporulation irrespective of inhibition of growth. Sporulation-inhibiting agents should not be dismissed as antifungal agents as the inhibition of sporulation may also be important for the control of fungal growth as spores are the main mode of transport of fungal growth and are linked to respiratory symptoms in the indoor air environment. Tea tree oil was the most effective antifungal agent tested, and may have industrial application for the remediation of fungal contamination in residential and occupational buildings. Further studies seem warranted to investigate the application of TTO and its growth inhibition efficacy on a wider range of fungal genera found in the indoor air environment. Antifungal agents should also be tested on contaminated surfaces, such as construction materials found within a building, to determine their activity. This would enable effective antifungal agents to be prescribed for application in indoor air quality control of residential and occupational buildings.
